# mTOR/miR-142-3p/PRAS40 signaling cascade is critical for tuberous sclerosis complex-associated renal cystogenesis

**DOI:** 10.1186/s11658-024-00638-x

**Published:** 2024-09-27

**Authors:** Shuyun Zhao, Shuai Hao, Jiasheng Zhou, Xinran Chen, Tianhua Zhang, Zhaolai Qi, Ting Zhang, Sajid Jalal, Chuanxin Zhai, Lu Yin, Yufei Bo, Hongming Teng, Yue Wang, Dongyan Gao, Hongbing Zhang, Lin Huang

**Affiliations:** 1 Department of Pathophysiology, College of Basic Medical Sciences, Dalian Medical University, Dalian, 116044 Liaoning People’s Republic of China; 2 Liaoning Provincial Key Laboratory of Medical Cellular and Molecular Biology, Dalian Medical University, Dalian, 116044 Liaoning People’s Republic of China; 3 Department of Pharmacology, Dalian Medical University, Dalian, 116044 Liaoning People’s Republic of China; 4 State Key Laboratory of Common Mechanism Research for Major Diseases, Haihe Laboratory of Cell Ecosystem, Department of Physiology, Institute of Basic Medical Sciences and School of Basic Medicine, Chinese Academy of Medical Sciences and Peking Union Medical College, Beijing, 100005 People’s Republic of China

**Keywords:** Tuberous sclerosis complex, mTOR, PRAS40, Renal cystogenesis, miRNA

## Abstract

**Background:**

Patients with tuberous sclerosis complex (TSC) develop renal cysts and/or angiomyolipomas (AMLs) due to inactive mutations of either *TSC1* or *TSC2* and consequential mTOR hyperactivation. The molecular events between activated mTOR and renal cysts/AMLs are still largely unknown.

**Methods:**

The mouse model of TSC-associated renal cysts were constructed by knocking out *Tsc2* specifically in renal tubules (*Tsc2*^f/f^; *ksp-Cre*). We further globally deleted PRAS40 in these mice to investigate the role of PRAS40. *Tsc2*^−/−^ cells were used as mTOR activation model cells. Inhibition of DNA methylation was used to increase miR-142-3p expression to examine the effects of miR-142-3p on PRAS40 expression and TSC-associated renal cysts.

**Results:**

PRAS40, a component of mTOR complex 1, was overexpressed in *Tsc2*-deleted cell lines and mouse kidneys (*Tsc2*^f/f^; *ksp-Cre*), which was decreased by mTOR inhibition. mTOR stimulated PRAS40 expression through suppression of miR-142-3p expression. Unleashed PRAS40 was critical to the proliferation of *Tsc2*^−/−^ cells and the renal cystogenesis of *Tsc2*^f/f^; *ksp-Cre* mice. In contrast, inhibition of DNA methylation increased miR-142-3p expression, decreased PRAS40 expression, and hindered cell proliferation and renal cystogenesis.

**Conclusions:**

Our data suggest that mTOR activation caused by TSC2 deletion increases PRAS40 expression through miR-142-3p repression. PRAS40 depletion or the pharmacological induction of miR-142-3p expression impaired TSC2 deficiency-associated renal cystogenesis. Therefore, harnessing mTOR/miR-142-3p/PRAS40 signaling cascade may mitigate hyperactivated mTOR-related diseases.

**Supplementary Information:**

The online version contains supplementary material available at 10.1186/s11658-024-00638-x.

## Introduction

Mechanistic target of rapamycin (mTOR) signaling cascade plays crucial roles in various physiological processes including cell survival, proliferation, and differentiation. This pathway is frequently dysregulated in many human diseases, including tuberous sclerosis complex (TSC) [1–3]. TSC is an autosomal dominant inherited multiorgan disorder manifesting renal angiomyolipoma (AMLs) and cysts, brain subependymal giant cell astrocytomas (SEGAs), malformations of the cerebral cortex (tubers), cardiac rhabdomyomas, pulmonary lymphangioleiomyomatosis (LAM), and so on. This disease is caused by inactivating mutations of either *TSC1* or *TSC2* and subsequent activation of mTOR signaling [4–8]. The first generation of mTOR inhibitors, the macrolide rapamycin (sirolimus) and its analogue everolimus (RAD001), have become standard therapy for TSC [9, 10]. However, cytostatic nature of rapalogs due to reactivation of AKT/protein kinase B (PKB) and mitogen-activated protein kinase (MAPK) warrants novel therapeutic strategies [11–13].

mTOR forms two distinct complexes, mTOR complex 1 (mTORC1) and mTORC2. As a component of mTORC1, PRAS40 (encoded by *AKT1S1*) is phosphorylated at Serine 183 by mTOR [14–18]. Elucidating the role of PRAS40 in mTOR signaling pathway may offer novel insights into the diseases with abnormal mTOR signaling.

mTOR is capable of regulating miRNA expression to fulfill its function [19, 20]. miR-142-3p modulates the proliferation and differentiation of hematopoietic cells and inhibits the formation and development of cancer [21–23]. Serum miRNA profiling in patients with TSC revealed a decline in miRNA levels, including miR-142-3p. Patients with TSC treated with everolimus had elevated serum miR-142-3p [24]. Whether miR-142-3p and its targets are involved in mTOR signaling transduction and TSC development is unknown.

In this study, we discovered the mTOR/miR-142-3p/PRAS40 signaling cascade and evaluated its significance in TSC2 deficiency-induced renal cyst formation. PRAS40 was increased while miR-142-3p was decreased in TSC2-deleted tissues and cell lines. The pharmacological induction of miR-142-3p expression or depletion of PRAS40 impaired renal cystogenesis caused by active mTOR. Therefore, targeting miR-142-3p/PRAS40 may treat active mTOR-related diseases.

## Materials and methods

### Mice

*Tsc2*^f/f^ and *ksp-Cre* mice were obtained from the Jackson Laboratory. *Tsc2*^f/f^; *Cre* mice were generated by intercrossing *Tsc2*^f/+^; *Cre* mice. *Akt1s1*^+*/*+^*Tsc2*^f/f^; *Cre* and *Akt1s1*^*−/−*^*Tsc2*^f/f^; *Cre* mice were obtained by serial intercrossing *Akt1s1*^*−/−*^*Tsc2*^f/+^; *Cre* with *Akt1s1*^+*/*+^*Tsc2*^f/+^; *Cre* mice.

The protocol of animal experiments was approved by the Animal Care and Ethics Committee of Dalian Medical University (AEE22083). All animal maintenance and procedures were carried out following the recommendations by the Animal Care and Ethics Committee of Dalian Medical University. All efforts were made to minimize suffering of mice. Mice were housed in a 12-h light-dark cycle and fed with a standard chow.

### Decitabine treatment

The *Tsc2*^f/f^; *Cre* male mice were injected intraperitoneally with decitabine at either 0.02 or 0.1 mg/kg/0.1 mL or Phosphate-buffered saline (PBS) at 0.1 mL/kg once every 3 days from postnatal day 11. All the mice were euthanized on postnatal day 20, and the kidneys were collected for analysis.

### Cell lines and cell culture

*Tsc2*^*−/−*^, *Tsc2*^+*/*+^, and *Pten*^*−/−*^ mouse embryonic fibroblasts (MEFs) have been described previously [11]; HEK293T cells (3101HUMGNHu17) were obtained from National Collection of Authenticated Cell Cultures, China and they were grown in Dulbecco’s Modified Eagle’s medium (DMEM); HK-2, NRK, and 786-0 cells were obtained from American Type Culture Collection (ATCC). NRK cells were grown in DMEM, and 786-0 cells were grown in Roswell Park Memorial Institute (RPM)I 1640, containing 10% fetal bovine serum (FBS) at 37 °C with 5% CO_2_. The cells were checked free of mycoplasma contamination by polymerase chain reaction (PCR), and were cultured for less than 6 months after resuscitation.

### Antibodies

Antibodies were purchased for detection of PRAS40, p-AKT (S473), S6, p-S6, TSC2, mTOR, Proliferating cell nuclear antigen (PCNA) (cell signaling), AKT, α-tubulin, β-actin, DNA methyltransferase 1 (DNMT1), and poly ADP-ribose polymerase (PARP) (Proteintech).

### Plasmids

PRAS40 expression vector was constructed by inserting the full length of mouse PRAS40 cDNA at the N-terminus into *Xba *I-*Bam*H I site of pCDH-CMV-MCS-EF1 vector. pLKO.1-mPRAS40 (Addgene plasmid #15480) was a gift from Dr. Do-Hyung Kim [17]; mTOR-1 shRNA (Addgene plasmid #1855), and mTOR-2 shRNA (Addgene plasmid #1856) were gifts from Dr. David Sabatini [25]. The construction of pCIneo-Luc-PRAS40 was described previously [26]. The binding site of miR-142-3p seed sequence in PRAS40’s 3'UTR was mutated from 5′-ACACTAC-3′ to 5′-AAAAAAA-3′. pCIneo-Luc-PRAS40 mutant was constructed by inserting the mutated PRAS40’s 3'UTR into the *Mlu *I-*Sal *I site of pCIneo-Luc.

### Oligonucleotide transfection

miR-142-3p mimics (4464066), inhibitor (4464084), and negative control miRNA (4464058) were synthesized by Thermo fisher. Oligonucleotide transfection was performed with Lipofectamine RNAiMAX reagent (Thermo fisher) following the company’s instructions.

### Lentivirus production and transduction

Virus particles were harvested 48 h after the expression vector transfection with packaging plasmid psPAX2 (gift from Dr. Didier Trono, Addgene plasmid #12260) and envelope plasmid pMD2.G (gift from Dr. Didier Trono, Addgene plasmid #12259) into HEK293T cells using Lipofectamine 2000 reagent (Thermo fisher). Cells were infected with lentivirus with the presence of 8 µg/mL polybrene (Sigma).

### Cell viability assay

Cells were seeded into 96-well plates in triplicate. Cell viability was determined by using Cell Counting Kit-8 (Dojindo, Kumamoto, Japan) at the indicated timepoints after treatment.

### Cystic index analysis

Hematoxylin and eosin (HE)-stained kidney sections were scanned in Pannoramic MIDI (3DHISTECH), and Image J software was used to quantify the area of cysts and kidney sections. Cystic indexes were obtained by calculating the percentages of the cystic areas in the areas of the entire kidney sections.

### Blood urea nitrogen (BUN) analysis

Mouse serum was applied to the BUN analysis using urea assay kit following the manufacturer’s instructions (C013-2–1, Nanjing Jiancheng Bioengineering Institute), absorbance was measured at 640 nm, and BUN concentration was calculated as: $\left( {{\text{testOD - blankOD}}} \right){\text{/}}\left( {{\text{standardOD - blankOD}}} \right)\,\, \times \,{\text{standard}}\,{\text{sample's}}\,{\text{concentration}}\,\left( {{\text{10}}\,{\text{mM}}} \right)$

### Real-time PCR analysis

Total RNA was isolated using Trizol reagent (Invitrogen), and cDNA was synthesized with the 5X All-In-One MasterMix (ABM). Quantitative real-time PCR analysis was performed in triplicate on LightCycler 480 Instrument (Roche) using TranStart Top Green qPCR SuperMix (Transgen Biotech). Threshold cycle (Ct) values of genes were normalized to those of β-actin. The following primers were used: PRAS40 FW: GATCGTCAGATG AGGAGAATGGC; PRAS40 RV: TGGAAGTCGCT GGTATTGAGCC; β-actin FW: CATTGCTGACAGGATGCAGAAGG; β-actin RV: TGCTGGAAGGTGGACAGTGAGG.

For miR-142-3p, cDNA was synthesized with TaqMan MicroRNA reverse transcription kit (Thermo). Quantitative real-time PCR analysis was performed in triplicate using TaqMan MicroRNA assays for miR-142-3p and TaqMan Universal Master Mix (Thermo).

### Immunoblotting analysis

Cells or tissues were lysed in lysis buffer [50 mM Tris (pH 7.4), 150 mM NaCl, 20 mM Na_2_HPO_4_/NaH_2_PO_4_, 10% glycerol, 1% Triton X-100, complete protease inhibitors and phosphatase inhibitors (Thermo)]. Total lysates were next applied to SDS-PAGE and then transferred onto PVDF membranes (Millipore). The membranes were blocked in 5% milk or 3% Bovine Serum Albumin (BSA)/ Tris Buffered Saline with Tween-20 (TBST). All the primary antibodies for western blot analysis were diluted in 3% BSA/TBST. HRP-conjugated secondary antibodies were diluted 1:10,000 in 5% milk/TBST, and the signals were detected with enhanced chemiluminescence (ECL) (Thermo).

### Immunohistochemistry (IHC)

Formalin-fixed, paraffin-embedded kidney sections were deparaffinized, dehydrated, and incubated with 0.3% hydrogen peroxide. Sections were incubated with anti-PCNA antibody overnight at 4 °C followed by incubation with biotinylated secondary antibodies (ZSGB-BIO, China) for 1 h at room temperature. Signals were detected with a diaminobenzidine substrate kit (ZSGB-BIO, China), followed by the counterstaining with hematoxylin.

Quantification of PCNA positive cells: calculate the percentages of PCNA-positive cells among the epithelial cells of the renal cysts. The average of three fields from each section, three sections for each group totally was calculated.

### Luciferase assay

HeLa cells were inoculated in 96-well plates, and transfected in triplicate with or without 10 nM of nonspecific control miRNA, miR-142-3p mimics, or miR-142-3p inhibitor, together with 200 ng/mL of pCIneo-Luc or pCIneo-Luc-PRAS40 or pCIneo-Luc-PRAS40 mutant using Lipofectamine 2000 (Invitrogen). Co-transfection with 20 ng/mLof phRL-SV40 (Promega) was used as the internal control. Luciferase activities were measured 48 h later.

### Statistical analyses

Data were presented as mean ± standard deviation (SD). Differences between groups were assessed by Student’s *t*-test or one-way analysis of variance (ANOVA). All experiments were repeated thrice. GraphPad Prism 7.04 software was used for all statistical analyses. *P* < 0.05 was considered statistically significant.

## Results

### PRAS40 expression is upregulated by mTOR activation

**Fig. 1:**
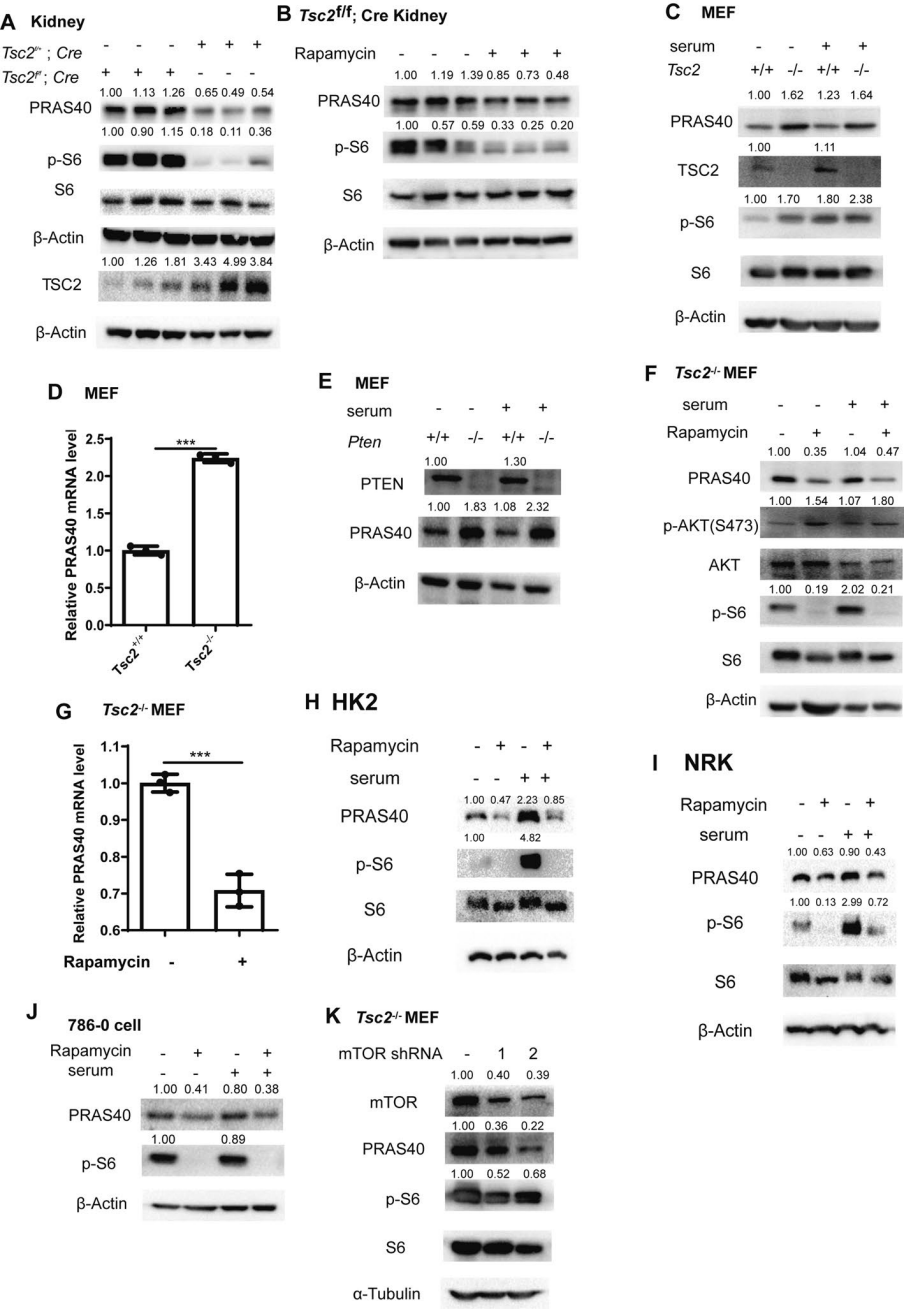
Association of PRAS40 expression with mTOR activation. Immunoblotting (**A**–**C**, **E**, **F**, **H**–**K**) or real-time PCR analysis (**D**, **G**). Tissue lysates were extracted from kidneys of *Tsc2*^f/+^; *ksp-cre* and *Tsc2*^f/f^; *ksp-cre* mice (**A**) or kidneys of *Tsc2*^f/f^; *ksp-cre* mice treated with or without rapamycin (**B**). *Tsc2*^+/+^ or *Tsc2*^−/−^ MEFs were cultured in the media with (+) or without serum (−) (**C**, **D**). *Pten*^−/−^ MEFs were cultured in the media with (+) or without serum (−) (**E**). *Tsc2*^−/−^ MEFs (**F**, **G**), HK2 (**H**), NRK (**I**), and 786–0 (**J**) cells were treated with or without rapamycin (5 nM) for 24 h. *Tsc2*^−/−^ MEFs were transfected with control shRNA, mTOR shRNA1, or mTOR shRNA2 (**K**). The quantification values of the band density were labeled above the bands. Bars, SD. ***, *P* < 0.001 from Student’s *t*-test

To delineate the molecular events between loss of TSC2 and the development of renal cysts of TSC, we first generated TSC cystic kidney model mice by crossing *Tsc2*^flox/flox^ mice (*Tsc2*^f/f^) with the mice that express Cre recombinase under the control of mouse *cadherin 16* (*Cdh16*) promoter (also known as *ksp-cre*), whose expression is limited to the renal tubules. As previously reported, the mice with deletion of *Tsc2* (*Tsc2*^f/f^; *Cre*) exhibited enlarged kidneys with poly-cysts and increased ratios of kidney weight to body weight (Supplementary Fig. 1A–D) [27–30]. These mice died between 35 and 60 days after birth. PRAS40 expression in renal tubules, but not the other organs of *Tsc2*^f/f^; *Cre* mice, was much higher than that in the kidneys of *Tsc2*^f/+^; *Cre* mice. Elevated PRAS40 was reduced by mTOR inhibitor rapamycin (Fig. 1A, B, Supplementary Fig. 1E, F). These data suggest an induction of PRAS40 expression by active mTOR.

Loss of TSC1, TSC2, or PTEN induces mTOR hyperactivation. Paired immortalized mouse embryonic fibroblast (MEF) cell lines deficient of these tumor suppressors have been widely used as mTOR activation model cell lines [4, 31–42]. To further clarify the relationship between PRAS40 and mTOR, we compared the levels of PRAS40 in *Tsc2*^+/+^ and *Tsc2*^−/−^ MEFs. Both mRNA and protein of PRAS40 were increased in *Tsc2*^−/−^ MEFs (Fig. 1C, D). A similar tendency was observed in *Pten*^−/−^ MEFs (Fig. 1E). Furthermore, rapamycin decreased both mRNA and protein of PRAS40 in *Tsc2*^−/−^ MEFs (Fig. 1F, G). Rapamycin also reduced PRAS40 in kidney cells HK2 and NRK, and renal cancer cell 786–0 (Fig. 1H–J). Similarly, mTOR shRNA decreased PRAS40 in *Tsc2*^−/−^ MEFs (Fig. 1K). Thus, mTOR activation leads to PRAS40 hyperexpression.

### PRAS40 is crucial to TSC-associated renal cystogenesis in mice

**Fig. 2:**
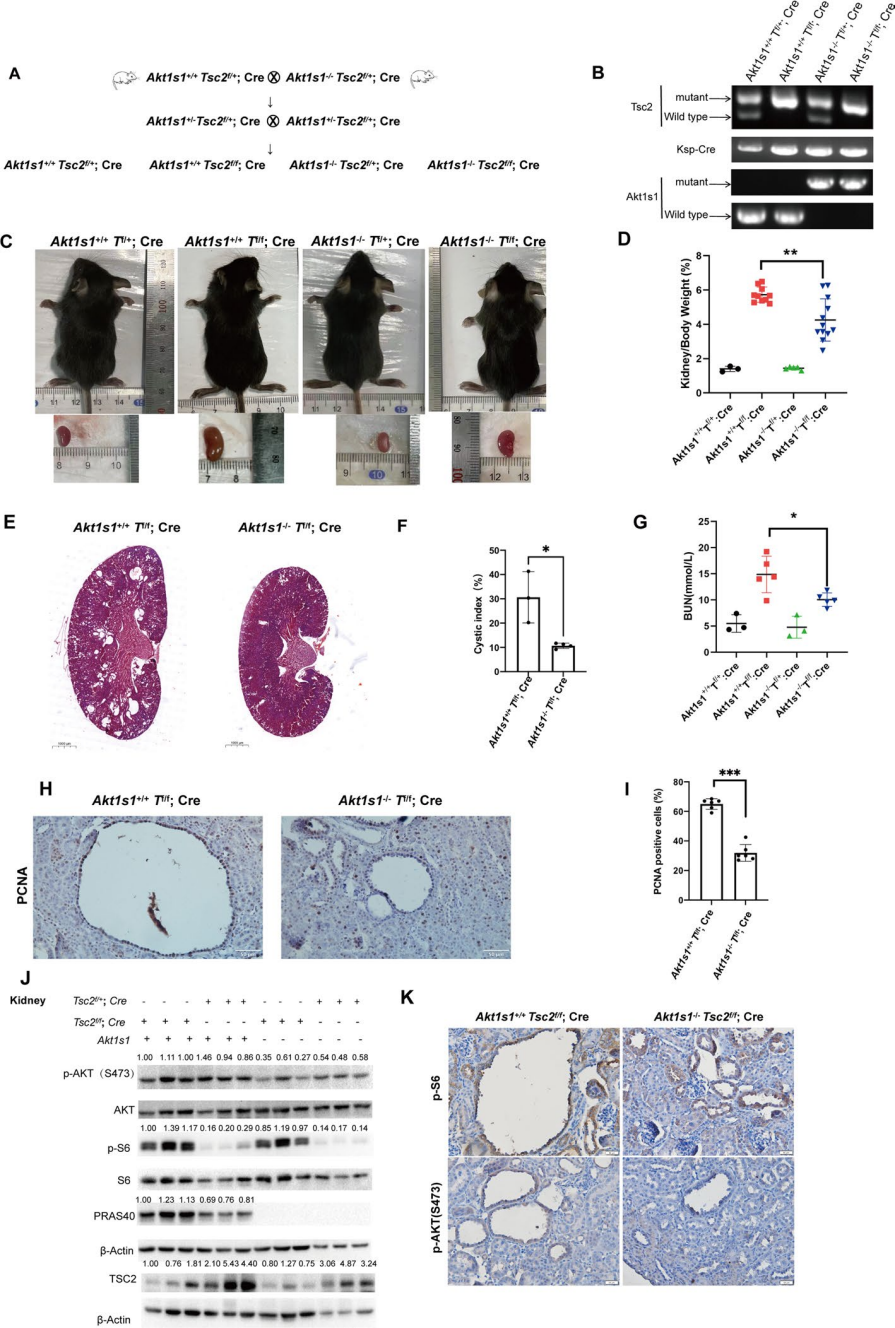
*Akt1s1* deletion impairs renal manifestations of *Tsc2*-deleted male mice. **A**. Mating strategy of the mice. **B**. Genotyping results. **C**. Images of male mice and their kidneys. **D**. Ratios of kidney weight to body weight. *n* = 3 for *Akt1s1*^+*/*+^*Tsc2*^f/+^; *Cre* mice; *n* = 10 for *Akt1s1*^+*/*+^*Tsc2*^f/f^; *Cre* mice; *n* = 5 for *Akt1s1*^*−/−*^*Tsc2*^f/+^; *Cre* mice; *n* = 12 for *Akt1s1*^*−/−*^*Tsc2*^f/f^; *Cre* mice. **E**. Hematoxylin and eosin (HE) staining of the kidneys. Scale bars, 1000 µm. **F**. Cystic indexes. *n* = 3 for *Akt1s1*^+*/*+^*Tsc2*^f/f^; *Cre* mice; *n* = 4 for *Akt1s1*^*−/−*^*Tsc2*^f/f^; *Cre* mice. **G**. BUN values. *n* = 3 for *Akt1s1*^+*/*+^*Tsc2*^f/+^; *Cre* and *Akt1s1*^*−/−*^*Tsc2*^f/+^; *Cre* mice; *n* = 5 for *Akt1s1*^+*/*+^*Tsc2*^f/f^; *Cre* and *Akt1s1*^*−/−*^*Tsc2*^f/f^; *Cre* mice. **H**. IHC staining with anti-PCNA antibody. Scale bars, 50 µm. **I**. Percentages of the PCNA positive cells in the epithelial cells of the cysts. **J**. Immunoblotting analysis for the kidneys. **K**. IHC staining with the indicated antibodies. Scale bars, 50 µm. The quantification values of the band density were labeled above the bands. Bars, SD. *P < 0.05, **P < 0.01, ***P < 0.001 from Student’s ***t***-test or one-way ANOVA

Renal PRAS40 expression was increased in *Tsc2*^f/f^; *Cre* mice but not in heterozygous mice (Fig. 1A, B). *Tsc2*^f/f^; *Cre* mice developed renal cysts (Supplementary Fig. 1). To investigate the role of PRAS40 in TSC-associated renal cysts, we crossed *Tsc2*^f/f^; *Cre* mice with *Akt1s1* globally deleted mice and obtained *Akt1s1*^*−/−*^*Tsc2*^f/f^; *Cre* mice (Fig. 2A, B). Compared with *Akt1s1*^+*/*+^*Tsc2*^f/f^; *Cre* mice, *Akt1s1*^*−/−*^*Tsc2*^f/f^; *Cre* mice exhibited smaller kidneys, lower ratios of kidney weight to body weight, fewer and smaller renal cysts, and lower cyst index (Fig. 2C–F, Supplementary Fig. 2). Moreover, *Akt1s1* deletion improved the kidney function manifested as reduction of blood urea nitrogen (BUN) in *Tsc2*^f/f^; *Cre* mice (Fig. 2G). As enhanced proliferation of cyst epithelial cells contributes to cyst formation [28], we stained kidney specimens with the antibody of PCNA, a proliferative marker. *Akt1s1* deletion reduced the number of PCNA-positive cells in renal cyst epithelial cells (Fig. 2H, I). Simultaneously, *Akt1s1* deletion suppressed the phosphorylation of AKT in *Tsc2*-deleted kidneys (Fig. 2J, K). These data suggest that PRAS40 is not only a downstream effector of mTOR signaling pathway, but also a promoter of AKT signaling.

### PRAS40 contributes to the cell proliferation induced by mTOR activation

**Fig. 3:**
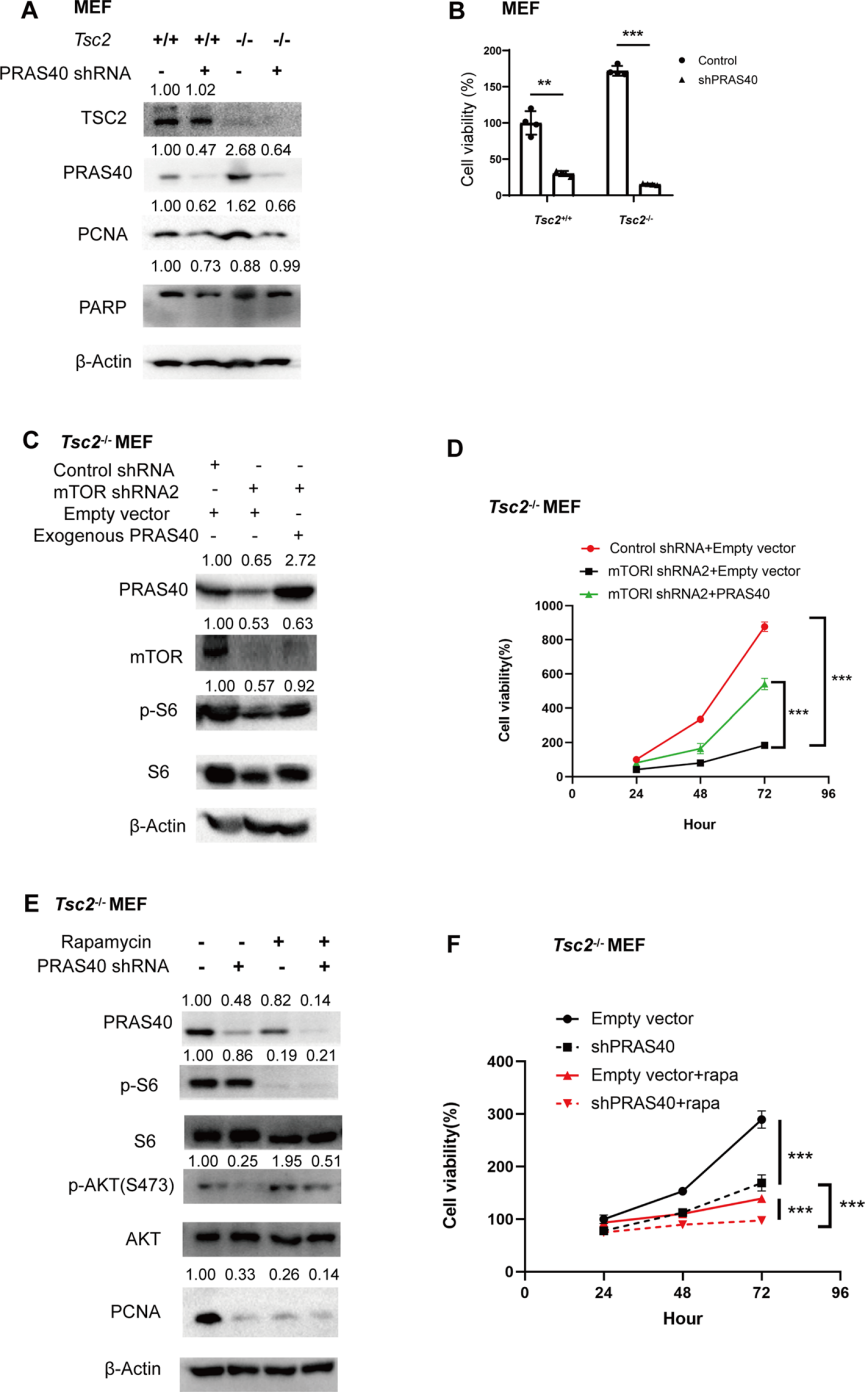
Influence of PRAS40 on the cell viability activated by mTOR. Immunoblotting (**A**, **C**, **E**) or cell viability assay (**B**, **D**, **F**). *Tsc2*^+/+^ or *Tsc2*^−/−^ MEFs transfected with control shRNA or PRAS40 shRNA (**A**, **B**). *Tsc2*^−/−^ MEFs were transfected with control shRNA, mTOR shRNA2, or mTOR shRNA2 together with empty vector or PRAS40 expression vector (**C**, **D**). *Tsc2*^−/−^ MEFs transfected with control shRNA or PRAS40 shRNA were treated with or without rapamycin (rapa, 5 nM) (**E**, **F**). The quantification values of the band density were labeled above the bands. Bars, SD. *P < 0.05, **P < 0.01, ***P < 0.001 from one-way ANOVA

We and others reported that PRAS40 promotes tumor cell proliferation [26, 43–45]. Given that PRAS40 depletion decreased PCNA expression in *Tsc2*-depleted renal cysts (Fig. 2), we checked the significance of PRAS40 in the proliferation of *Tsc2*^−/−^ MEFs. PRAS40 depletion with shRNA exerted greater suppression on the viability of *Tsc2*^−/−^ MEFs than on that of *Tsc2*^+/+^ MEFs. Furthermore, PCNA was decreased in shPRAS40-treated cells, whereas apoptosis marker cleaved-PARP was not detected in any of the samples (Fig. 3A, B). In addition, mTOR depletion inhibited PRAS40 expression and cell viability, which were reversed by PRAS40 overexpression (Fig. 3C, D). Moreover, combination of rapamycin and PRAS40 shRNA was superior in suppression of cell proliferation to rapamycin or PRAS40 shRNA alone. AKT phosphorylation induced by rapamycin was also suppressed by PRAS40 depletion (Fig. 3E, F). Therefore, PRAS40 is critical for mTOR-potentiated cell proliferation. Targeting PRAS40 may alleviate rapamycin-induced reactivation of AKT and enhance the efficacy of rapamycin.

### miR-142-3p reduces PRAS40 abundance through targeting its 3'UTR

**Fig. 4:**
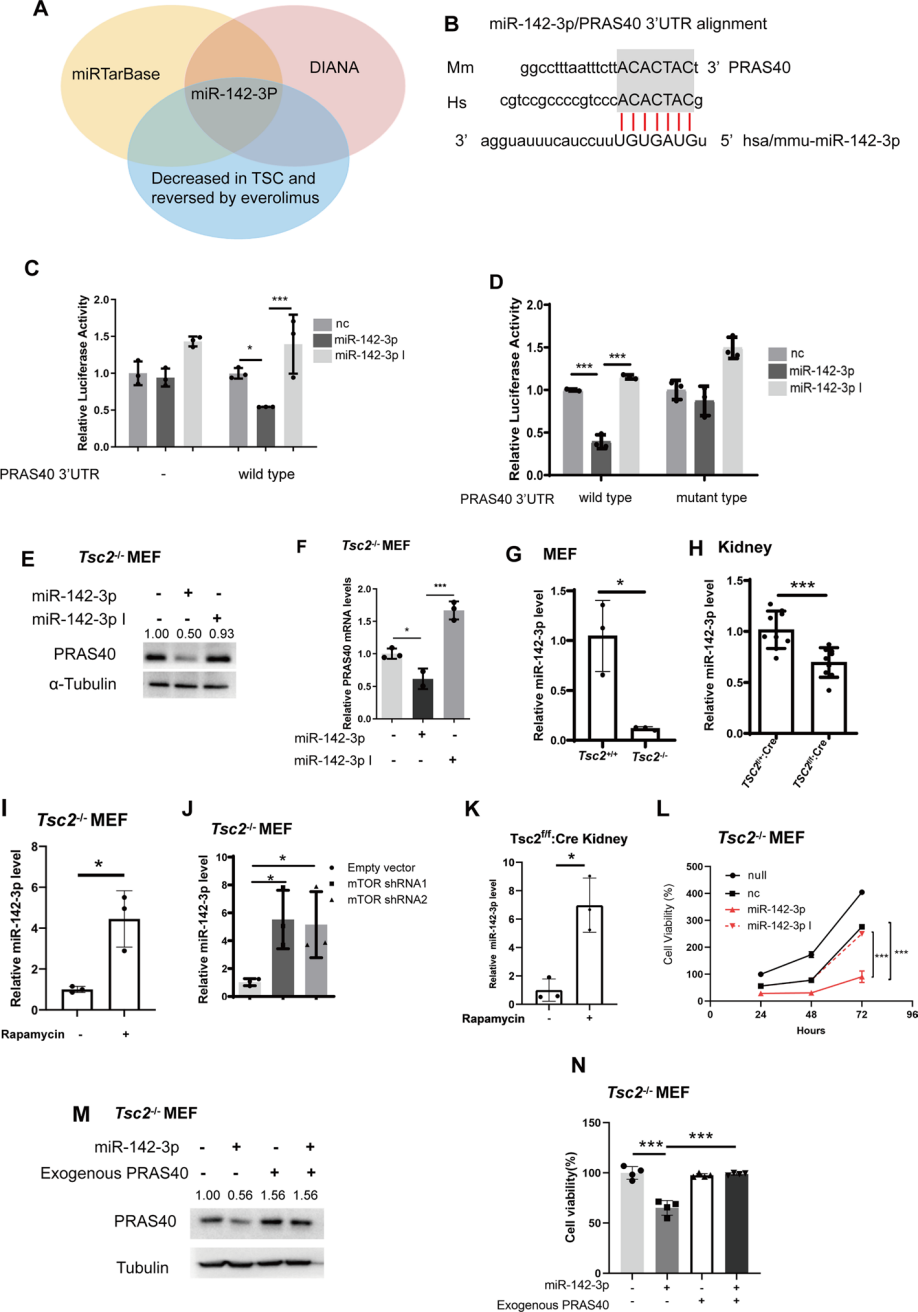
Association of miR-142-3p expression with mTOR activation and PRAS40 expression. **A**. Prediction of the miRNAs binding to PRAS40 3'UTR in those decreased in patients with TSC. **B**. Alignment of miR-142-3p to PRAS40’s 3'UTR. **C**, **D**. Luciferase assays. pCIneo-Luc (−), pCIneo-Luc-PRAS40 (wild type), or pCIneo-Luc-PRAS40 mutant (mutant type) reporter vector and nonspecific control miRNA (nc), miR-142-3p mimic (miR-142-3p) or miR-142-3p inhibitor (miR-142-3p I) together with *Renilla* luciferase expression vector phRL-SV40 were cotransfected into cells. The experiments were performed in triplicate. **E**, **F**. *Tsc2*^−/−^ MEFs were transfected with nonspecific control miRNA (−), miR-142-3p mimic (miR-142-3p) or miR-142-3p inhibitor (miR-142-3p I). Immunoblotting (**E**) and real-time PCR analysis (**F**) were performed. **G–K**. Real-time PCR analysis. *Tsc2*^+/+^ or *Tsc2*^−/−^ MEFs were cultured in serum free media for 24 h (**G**). Kidney tissues from *Tsc2*^f/f^; *ksp-cre* or *Tsc2*^f/+^; *ksp-cre* mice (**H**). *Tsc2*^−/−^ MEFs were treated with or without rapamycin (5 nM) for 24 h (**I**). *Tsc2*^−/−^ MEFs were transfected with control shRNA, mTOR shRNA1, or mTOR shRNA2 (**J**). Kidney tissues from *Tsc2*^f/f^; *ksp-cre* treated with or without rapamycin (**K**). **L**. Cell viability assay of *Tsc2*^−/−^ MEFs transfected without (−, null) or with nonspecific control miRNA (nc), miR-142-3p mimic (miR-142-3p) or miR-142-3p inhibitor (miR-142-3p I). **M–N**. *Tsc2*^−/−^ MEFs were cotransfected with negative control (−) or miR-142-3pmimic (miR-142-3p) together with empty vector or PRAS40 expression vector. PRAS40 protein level was analyzed by Immunoblotting analysis (**M**), and cell viability assays were performed (**N**). The quantification values of the band density were labeled above the bands. Bars, SD. *P < 0.05, **P < 0.01, ***P < 0.001 from Student’s *t*-test or one-way ANOVA

To investigate the mechanism underlying mTOR activation of PRAS40 expression, we explored the potential role of miRNAs. Using both miRTarBase and DIANA-TarBase, we predicted the miRNAs which might bind PRAS40 3'UTR and then focused on those that are downregulated in the plasma of patients with TSC but are reversed by everolimus treatment [24]. In the end, miR-142-3p was selected as the most promising candidate (Fig. 4A). miR-142-3p binding site within the PRAS40's 3'UTR is conserved between human and mouse (Fig. 4B). Compared with negative control miRNA, miR-142-3p mimic but not miR-142-3p inhibitor reduced the luciferase activity of the reporter containing PRAS40's 3'UTR (wild type). The luciferase activity of the control reporter without PRAS40's 3'UTR (−) was not altered by miR-142-3p (Fig. 4C). Furthermore, neither miR-142-3p mimic nor the inhibitor affected the luciferase activity of the reporter including PRAS40's 3'UTR with the mutation of miR-142-3p binding site (mutant type) (Fig. 4D). To confirm the effects of miR-142-3p on PRAS40 expression, we introduced miR-142-3p into *Tsc2*^−/−^ MEFs. Compared with negative control miRNA, miR-142-3p mimic but not the inhibitor decreased both the mRNA and protein levels of PRAS40 (Fig. 4E, F). Therefore, miR-142-3p inhibits PRAS40 expression by targeting its 3'UTR.

### mTOR activation promotes cell proliferation through suppression of miR-142-3p

Plasma levels of miR-142-3p are downregulated in patients with TSC but are reversed by everolimus treatment [24]. The expression of miR-142-3p was indeed much lower in *Tsc2*^−/−^ MEFs than in *Tsc2*^+/+^ MEFs (Fig. 4G). In addition, miR-142-3p level in the kidneys of *Tsc2*^f/f^; *Cre* mice was also much lower than in that of *Tsc2*^f/+^; *Cre* mice (Fig. 4H). Moreover, rapamycin or mTOR shRNA increased miR-142-3p in *Tsc2*^−/−^ MEFs (Fig. 4I, J). Rapamycin also augmented miR-142-3p level in the kidneys of *Tsc2*^f/f^; *Cre* mice (Fig. 4K). Thus, mTOR activation leads to the downregulation of miR-142-3p.

To investigate the biological effect of miR-142-3p in mTOR activated cells, we transfected a miR-142-3p mimic into *Tsc2*^−/−^ MEFs. The cells transfected with the miR-142-3p mimic showed a notable decline in cell viability compared with the cells transfected with negative control miRNA, whereas the miR-142-3p inhibitor did not show any remarkable change (Fig. 4L). These data suggest that mTOR activation promotes cell proliferation by downregulating the expression of miR-142-3p.

To verify the relationship between miR-142-3p and PRAS40, we cotransfected miR-142-3p mimic together with PRAS40 into *Tsc2*^−/−^ MEFs. Since the expression plasmid contained PRAS40 coding DNA without the 3'UTR region of PRAS40, endogenous but not exogenous PRAS40 expression was suppressed by miR-142-3p (Fig. 4M). Further, miR-142-3p downregulated cell viability compared with the negative control miRNA, while PRAS40 overexpression revived the cells inhibited by miR-142-3p (Fig. 4N). Thus, miR-142-3p suppresses cell proliferation through downregulating PRAS40 expression.

### Inhibition of DNA methylation represses renal cystogenesis

**Fig. 5:**
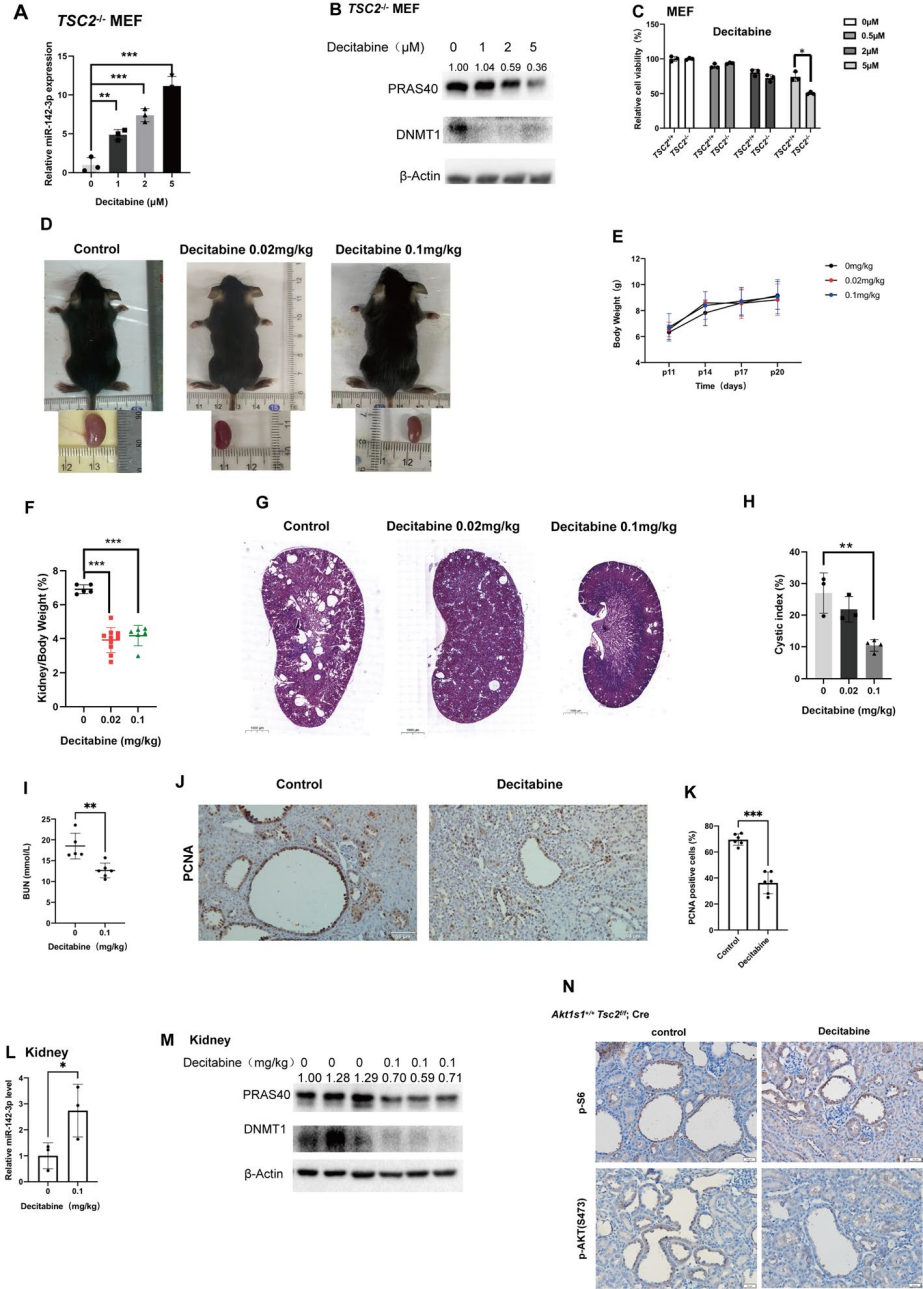
Effects of methylation inhibition in mice and MEFs. **A**–**C**. MEFs were treated with or without decitabine at indicated concentrations for 48 h. Real-time PCR (**A**), immunoblotting (**B**), and relative cell viability assay (**C**) were performed. **D–N**. *Tsc2*^f/f^; *Cre* mice were treated with decitabine or PBS (control). Images of the mice and the kidneys (**D**). Body weights of the mice from postnatal days 11–20. *n* = 5 for PBS-treated mice; *n* = 6 for decitabine (0.02 mg/kg)-treated mice; *n* = 10 for decitabine (0.1 mg/kg)-treated mice (**E**). Ratios of kidney weight to body weight (**F**). HE staining of the kidneys. Scale bars, 1000 µm (**G**). Cystic indexes. *n* = 3 for PBS-treated mice and decitabine (0.02 mg/kg)-treated mice; *n* = 4 for decitabine (0.1 mg/kg)-treated mice (**H**). BUN values. *n* = 5 for PBS-treated mice; *n* = 6 for decitabine (0.1 mg/kg)-treated mice (**I**). IHC staining with anti-PCNA antibody. Scale bars, 50 µm (**J**). Percentages of the PCNA-positive cells in the epithelial cells of the cysts (**K**). Real-time PCR analysis for the kidney tissues (*n* = 3) (**L**). Immunoblotting analysis for the kidneys (**M**). IHC staining with the indicated antibodies. Scale bars, 50 µm (**N**). The quantification values of the band density were labeled above the bands. Bars, SD. *P < 0.05, **P < 0.01, ***P < 0.001 from Student’s *t*-test or one-way ANOVA

Since DNA methylation is essential for miR-142-3p hypoexpression in proliferative cells [46–48], we treated *Tsc2*^−/−^ MEFs with DNA methylation inhibitor decitabine, which downregulated DNMT1 expression. As a consequence, an increase in miR-142-3p level and a decrease in PRAS40 level were observed. Meanwhile, the cell viability was repressed (Fig. 5A–C).

Next, we treated *Tsc2*^f/f^; *Cre* mice with various doses of decitabine. No significant toxicity was observed in the treated mice on the basis of changes in body weight. Compared with PBS treatment, decitabine caused smaller kidneys, lower ratios of kidney weight to body weight, reduced cyst indexes, and declined BUN levels in *Tsc2*^f/f^; *Cre* mice in a dose-dependent manner (Fig. 5D–I). The percentage of PCNA-positive cyst epithelial cells was also downregulated by decitabine (Fig. 5J, K). Accordingly, miR-142-3p level was upregulated and PRAS40 level was downregulated (Fig. 5L, M). AKT phosphorylation was decreased (Fig. 5N). Therefore, inhibition of DNA methylation blocks renal cystogenesis induced by mTOR activation.

## Discussion

**Fig. 6:**
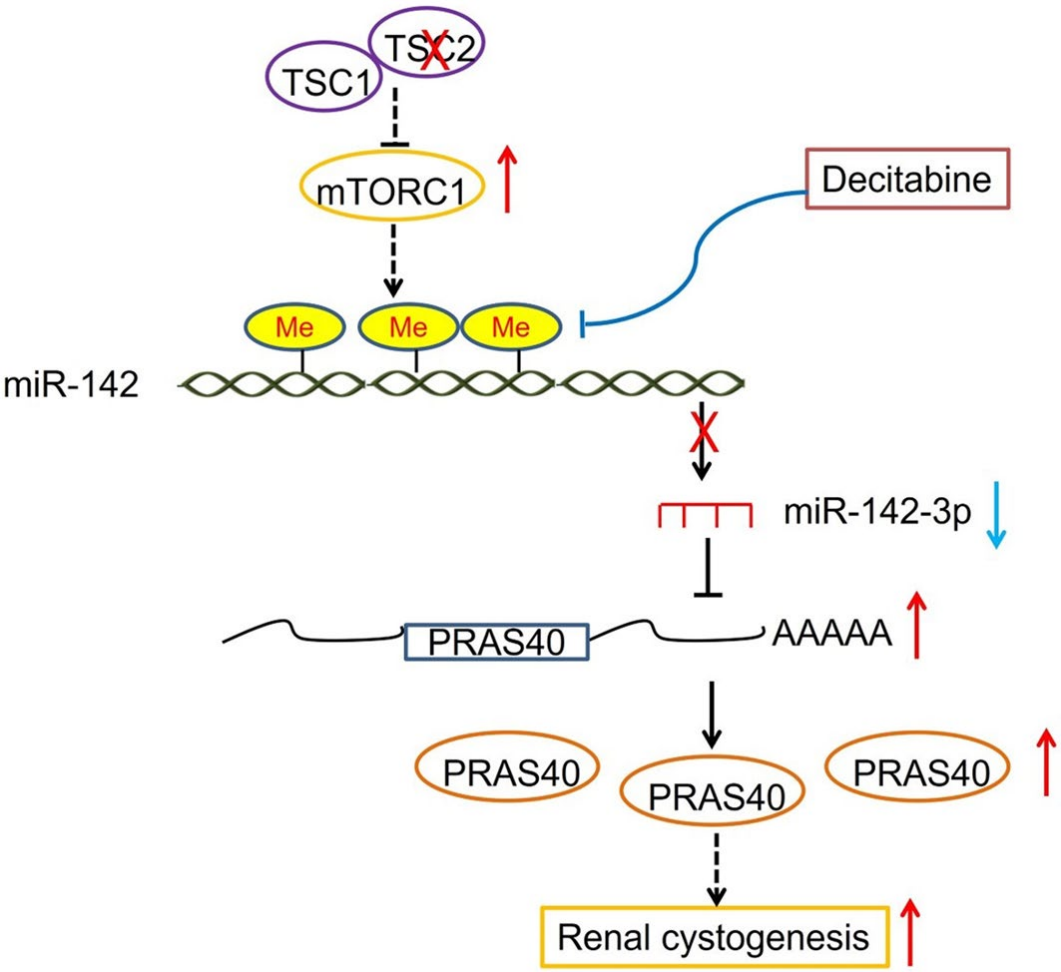
Schematic graph representing the pathogenesis of TSC-associated renal cystogenesis. Me, methyl. →, promotion; ┤, suppression

Abnormal mTOR signaling triggers numerous diseases including TSC. TSC-associated renal AMLs and cysts are the most common causes of death in patients with TSC [49, 50]. Here, we presented that mTOR activation led to renal cystogenesis by increasing PRAS40 expression through repression of miR-142-3p expression. Depletion of PRAS40 or increasing miR-142-3p expression via inhibition of DNA methylation could be potential therapeutic strategies for TSC-associated renal cysts (Fig. 6).

PRAS40 is a component of mTORC1 and is phosphorylated by mTOR [14–18]. It either negatively or positively regulates mTOR pathway signaling in a context-dependent manner [14, 16–18, 43, 51]. In this study, we found that mTOR activation increased mRNA and protein levels of PRAS40. Therefore, the regulation of PRAS40 by mTOR is multidimensional, including direct phosphorylation and indirect upregulation of PRAS40 expression. In addition, PRAS40 contributed to the proliferation of *Tsc2*^−/−^ MEFs and the renal cyst epithelial cells, and cystogenesis of *Tsc2*-deleted mouse kidneys. Therefore, PRAS40 is essential for fulfilling the function of mTOR signaling pathway in cell proliferation and renal cystogenesis. However, the phosphorylation of mTOR downstream factor S6 was not changed by PRAS40 deletion in *Tsc2*-deleted cells and kidneys, indicating that PRAS40 works either downstream of S6 or in parallel with S6. Nevertheless, these data suggest that PRAS40 can be targeted for TSC-associated cystogenesis.

Consistent with the effects of PRAS40 in tumor cells [17, 43, 44], PRAS40 promoted AKT phosphorylation in *Tsc2*-depleted MEFs and mouse kidneys. Since PRAS40 overexpression increased AKT phosphorylation caused by mTOR knockdown, PRAS40 stimulation of AKT phosphorylation should not be related to mTORC2 activation. Therefore, PRAS40 deletion inhibits cell proliferation and renal cystogenesis not only by hindering mTOR signaling, but also by suppressing AKT activation. Moreover, PRAS40 shRNA enhanced rapamycin inhibition of cell proliferation, likely by abolishing rapamycin-induced AKT phosphorylation. Thus, targeting PRAS40 could combine with mTOR inhibition for the treatment of mTOR hyperactivated diseases such as TSC.

The next question is how PRAS40 expression is upregulated by mTOR activation. mTOR signaling alters miRNA expression [19, 20]. miR-142-3p was decreased in serum of patients with TSC but restored to baseline level in control serum by treatment of mTOR inhibitor [24]. Our data showed that miR-142-3p was suppressed by mTOR activation. miR-142-3p suppresses cell proliferation by binding to PRAS40's 3'UTR and reducing PRAS40 expression. Thus, mTOR activation upregulates PRAS40 through inhibiting miR-142-3p. On the basis of the previous serum data and our MEF results, this mTOR/miR-142-3p/PRAS40 regulatory axis may extend beyond the kidney to other organs. Further investigation using additional TSC models including AML is warranted to confirm this hypothesis.

DNA methylation decreases miR-142-3p expression [46, 47]. Here, decitabine increased miR-142-3p expression and decreased PRAS40 expression in *Tsc2*^−/−^ MEFs and *Tsc2*-depleted kidneys. Consequently, decitabine inhibited renal cell proliferation and cystogenesis in *Tsc2*-deleted mice. These data not only confirmed the upregulation of PRAS40 by mTOR through suppression of miR-142-3p, but also provided a proof-of-concept of restoring miR-142-3p for treating TSC-associated renal cysts. Additionally, more specific strategies targeting the DNA methylation of miR-142-3p may offer more precise therapeutic intervention.

The mechanism underlying TSC-associated renal cystogenesis remains complex. Various research has revealed that downstream effectors of mTOR, such as TFEB and TFE3, play crucial roles in this disease [30, 52, 53]. PRAS40 is a novel one; future therapeutic strategies could consider targeting these factors in combination.

## Conclusions

In this study, we find that PRAS40 is a novel downstream effector of mTOR signaling pathway. Targeting PRAS40 directly or suppressing PRAS40 expression indirectly through enhancement of miR-142-3p impairs the formation of TSC-associated renal cysts, which may provide promising therapeutic approaches for diseases with hyperactivated mTOR signaling.

## Supplementary Information

Additional file 1.

## Data Availability

Not applicable.

## Abbreviations

PRAS40: Proline rich Akt substrate 40
TSC: Tuberous sclerosis complex
AML: Angiomyolipoma
mTOR: Mechanistic target of rapamycin
SEGA: Subependymal giant cell astrocytomas
LAM: Lymphangioleiomyomatosis
MEF: Mouse embryonic fibroblast
BUN: Blood urea nitrogen

## References

[1] Mossmann D, Park S, Hall MN. mTOR signalling and cellular metabolism are mutual determinants in cancer. Nat Rev Cancer. 2018;18:744–57. 30425336 10.1038/s41568-018-0074-8

[2] Panwar V, Singh A, Bhatt M, Tonk RK, Azizov S, Raza AS, et al. Multifaceted role of mTOR (mammalian target of rapamycin) signaling pathway in human health and disease. Signal Transduct Target Ther. 2023;8:375. 37779156 10.1038/s41392-023-01608-z PMC10543444

[3] Saxton RA, Sabatini DM. mTOR signaling in growth, metabolism, and disease. Cell. 2017;168:960–76. 28283069 10.1016/j.cell.2017.02.004 PMC5394987

[4] Kwiatkowski DJ, Zhang H, Bandura JL, Heiberger KM, Glogauer M, El-Hashemite N, et al. A mouse model of TSC1 reveals sex-dependent lethality from liver hemangiomas, and up-regulation of p70S6 kinase activity in Tsc1 null cells. Hum Mol Genet. 2002;11:525–34. 11875047 10.1093/hmg/11.5.525

[5] Manning BD, Tee AR, Logsdon MN, Blenis J, Cantley LC. Identification of the tuberous sclerosis complex-2 tumor suppressor gene product tuberin as a target of the phosphoinositide 3-kinase/akt pathway. Mol Cell. 2002;10:151–62. 12150915 10.1016/s1097-2765(02)00568-3

[6] Potter CJ, Pedraza LG, Xu T. Akt regulates growth by directly phosphorylating Tsc2. Nat Cell Biol. 2002;4:658–65. 12172554 10.1038/ncb840

[7] Inoki K, Li Y, Zhu T, Wu J, Guan KL. TSC2 is phosphorylated and inhibited by Akt and suppresses mTOR signalling. Nat Cell Biol. 2002;4:648–57. 12172553 10.1038/ncb839

[8] Gao X, Zhang Y, Arrazola P, Hino O, Kobayashi T, Yeung RS, et al. Tsc tumour suppressor proteins antagonize amino-acid-TOR signalling. Nat Cell Biol. 2002;4:699–704. 12172555 10.1038/ncb847

[9] Bissler JJ, McCormack FX, Young LR, Elwing JM, Chuck G, Leonard JM, et al. Sirolimus for angiomyolipoma in tuberous sclerosis complex or lymphangioleiomyomatosis. N Engl J Med. 2008;358:140–51. 18184959 10.1056/NEJMoa063564 PMC3398441

[10] McCormack FX, Inoue Y, Moss J, Singer LG, Strange C, Nakata K, et al. Efficacy and safety of sirolimus in lymphangioleiomyomatosis. N Engl J Med. 2011;364:1595–606. 21410393 10.1056/NEJMoa1100391 PMC3118601

[11] Zhang H, Bajraszewski N, Wu E, Wang H, Moseman AP, Dabora SL, et al. PDGFRs are critical for PI3K/Akt activation and negatively regulated by mTOR. J Clin Invest. 2007;117:730–8. 17290308 10.1172/JCI28984 PMC1784000

[12] Jahn SC, Law ME, Corsino PE, Davis BJ, Harrison JK, Law BK. Signaling mechanisms that suppress the cytostatic actions of rapamycin. PLoS ONE. 2014;9:e99927. 24927123 10.1371/journal.pone.0099927 PMC4057458

[13] Lu Y, Zhang EY, Liu J, Yu JJ. Inhibition of the mechanistic target of rapamycin induces cell survival via MAPK in tuberous sclerosis complex. Orphanet J Rare Dis. 2020;15:209. 32807195 10.1186/s13023-020-01490-w PMC7433150

[14] Fonseca BD, Smith EM, Lee VH, MacKintosh C, Proud CG. PRAS40 is a target for mammalian target of rapamycin complex 1 and is required for signaling downstream of this complex. J Biol Chem. 2007;282:24514–24. 17604271 10.1074/jbc.M704406200

[15] Oshiro N, Takahashi R, Yoshino K, Tanimura K, Nakashima A, Eguchi S, et al. The proline-rich Akt substrate of 40 kDa (PRAS40) is a physiological substrate of mammalian target of rapamycin complex 1. J Biol Chem. 2007;282:20329–39. 17517883 10.1074/jbc.M702636200 PMC3199301

[16] Sancak Y, Thoreen CC, Peterson TR, Lindquist RA, Kang SA, Spooner E, et al. PRAS40 is an insulin-regulated inhibitor of the mTORC1 protein kinase. Mol Cell. 2007;25:903–15. 17386266 10.1016/j.molcel.2007.03.003

[17] Vander Haar E, Lee SI, Bandhakavi S, Griffin TJ, Kim DH. Insulin signalling to mTOR mediated by the Akt/PKB substrate PRAS40. Nat Cell Biol. 2007;9:316–23. 17277771 10.1038/ncb1547

[18] Wang L, Harris TE, Roth RA, Lawrence JC Jr. PRAS40 regulates mTORC1 kinase activity by functioning as a direct inhibitor of substrate binding. J Biol Chem. 2007;282:20036–44. 17510057 10.1074/jbc.M702376200

[19] Gai X, Tang B, Liu F, Wu Y, Wang F, Jing Y, et al. mTOR/miR-145-regulated exosomal GOLM1 promotes hepatocellular carcinoma through augmented GSK-3beta/MMPs. J Genet Genomics. 2019;46:235–45. 31186161 10.1016/j.jgg.2019.03.013

[20] Liko D, Rzepiela A, Vukojevic V, Zavolan M, Hall MN. Loss of TSC complex enhances gluconeogenesis via upregulation of Dlk1-Dio3 locus miRNAs. Proc Natl Acad Sci USA. 2020;117:1524–32. 31919282 10.1073/pnas.1918931117 PMC6983401

[21] Sun W, Shen W, Yang S, Hu F, Li H, Zhu TH. miR-223 and miR-142 attenuate hematopoietic cell proliferation, and miR-223 positively regulates miR-142 through LMO2 isoforms and CEBP-beta. Cell Res. 2010;20:1158–69. 20856265 10.1038/cr.2010.134

[22] Isobe T, Hisamori S, Hogan DJ, Zabala M, Hendrickson DG, Dalerba P, et al. miR-142 regulates the tumorigenicity of human breast cancer stem cells through the canonical WNT signaling pathway. Elife. 2014. 10.7554/eLife.01977. 25406066 10.7554/eLife.01977 PMC4235011

[23] Li Y, He Q, Wen X, Hong X, Yang X, Tang X, et al. EZH2-DNMT1-mediated epigenetic silencing of miR-142-3p promotes metastasis through targeting ZEB2 in nasopharyngeal carcinoma. Cell Death Differ. 2019;26:1089–106. 30353102 10.1038/s41418-018-0208-2 PMC6748116

[24] Trelinska J, Fendler W, Dachowska I, Kotulska K, Jozwiak S, Antosik K, et al. Abnormal serum microRNA profiles in tuberous sclerosis are normalized during treatment with everolimus: possible clinical implications. Orphanet J Rare Dis. 2016;11:129. 27680012 10.1186/s13023-016-0512-1 PMC5041396

[25] Sarbassov DD, Guertin DA, Ali SM, Sabatini DM. Phosphorylation and regulation of Akt/PKB by the rictor-mTOR complex. Science. 2005;307:1098–101. 15718470 10.1126/science.1106148

[26] Huang L, Nakai Y, Kuwahara I, Matsumoto K. PRAS40 is a functionally critical target for EWS repression in Ewing sarcoma. Cancer Res. 2012;72:1260–9. 22241085 10.1158/0008-5472.CAN-11-2254

[27] Ren S, Luo Y, Chen H, Warburton D, Lam HC, Wang LL, et al. Inactivation of Tsc2 in mesoderm-derived cells causes polycystic kidney lesions and impairs lung alveolarization. Am J Pathol. 2016;186:3261–72. 27768862 10.1016/j.ajpath.2016.08.013 PMC5225296

[28] Drusian L, Nigro EA, Mannella V, Pagliarini R, Pema M, Costa ASH, et al. mTORC1 upregulation leads to accumulation of the oncometabolite fumarate in a mouse model of renal cell carcinoma. Cell Rep. 2018;24(1093–104):e6. 10.1016/j.celrep.2018.06.106 30067967

[29] Bonucci M, Kuperwasser N, Barbe S, Koka V, de Villeneuve D, Zhang C, et al. mTOR and S6K1 drive polycystic kidney by the control of Afadin-dependent oriented cell division. Nat Commun. 2020;11:3200. 32581239 10.1038/s41467-020-16978-z PMC7314806

[30] Pema M, Drusian L, Chiaravalli M, Castelli M, Yao Q, Ricciardi S, et al. mTORC1-mediated inhibition of polycystin-1 expression drives renal cyst formation in tuberous sclerosis complex. Nat Commun. 2016;7:10786. 26931735 10.1038/ncomms10786 PMC4778067

[31] Zhang Y, Nicholatos J, Dreier JR, Ricoult SJ, Widenmaier SB, Hotamisligil GS, et al. Coordinated regulation of protein synthesis and degradation by mTORC1. Nature. 2014;513:440–3. 25043031 10.1038/nature13492 PMC4402229

[32] Inoki K, Zhu T, Guan KL. TSC2 mediates cellular energy response to control cell growth and survival. Cell. 2003;115:577–90. 14651849 10.1016/s0092-8674(03)00929-2

[33] Hsu PP, Kang SA, Rameseder J, Zhang Y, Ottina KA, Lim D, et al. The mTOR-regulated phosphoproteome reveals a mechanism of mTORC1-mediated inhibition of growth factor signaling. Science. 2011;332:1317–22. 21659604 10.1126/science.1199498 PMC3177140

[34] Kovalenko A, Sanin A, Kosmas K, Zhang L, Wang J, Akl EW, et al. Therapeutic targeting of DGKA-mediated macropinocytosis leads to phospholipid reprogramming in tuberous sclerosis complex. Cancer Res. 2021;81:2086–100. 33593821 10.1158/0008-5472.CAN-20-2218 PMC8137542

[35] Alesi N, Akl EW, Khabibullin D, Liu HJ, Nidhiry AS, Garner ER, et al. TSC2 regulates lysosome biogenesis via a non-canonical RAGC and TFEB-dependent mechanism. Nat Commun. 2021;12:4245. 34253722 10.1038/s41467-021-24499-6 PMC8275687

[36] Lee DF, Kuo HP, Chen CT, Hsu JM, Chou CK, Wei Y, et al. IKK beta suppression of TSC1 links inflammation and tumor angiogenesis via the mTOR pathway. Cell. 2007;130:440–55. 17693255 10.1016/j.cell.2007.05.058

[37] Zhang H, Cicchetti G, Onda H, Koon HB, Asrican K, Bajraszewski N, et al. Loss of Tsc1/Tsc2 activates mTOR and disrupts PI3K-Akt signaling through downregulation of PDGFR. J Clin Invest. 2003;112:1223–33. 14561707 10.1172/JCI17222 PMC213485

[38] Ma J, Meng Y, Kwiatkowski DJ, Chen X, Peng H, Sun Q, et al. Mammalian target of rapamycin regulates murine and human cell differentiation through STAT3/p63/Jagged/Notch cascade. J Clin Invest. 2010;120:103–14. 20038814 10.1172/JCI37964 PMC2798675

[39] Sun Q, Chen X, Ma J, Peng H, Wang F, Zha X, et al. Mammalian target of rapamycin up-regulation of pyruvate kinase isoenzyme type M2 is critical for aerobic glycolysis and tumor growth. Proc Natl Acad Sci USA. 2011;108:4129–34. 21325052 10.1073/pnas.1014769108 PMC3054028

[40] Valvezan AJ, Turner M, Belaid A, Lam HC, Miller SK, McNamara MC, et al. mTORC1 couples nucleotide synthesis to nucleotide demand resulting in a targetable metabolic vulnerability. Cancer Cell. 2017;32(624–38):e5. 10.1016/j.ccell.2017.09.013 PMC5687294 29056426

[41] Howell JJ, Hellberg K, Turner M, Talbott G, Kolar MJ, Ross DS, et al. Metformin inhibits hepatic mTORC1 signaling via dose-dependent mechanisms involving AMPK and the TSC complex. Cell Metab. 2017;25:463–71. 28089566 10.1016/j.cmet.2016.12.009 PMC5299044

[42] Menon S, Dibble CC, Talbott G, Hoxhaj G, Valvezan AJ, Takahashi H, et al. Spatial control of the TSC complex integrates insulin and nutrient regulation of mTORC1 at the lysosome. Cell. 2014;156:771–85. 24529379 10.1016/j.cell.2013.11.049 PMC4030681

[43] Lv D, Liu J, Guo L, Wu D, Matsumoto K, Huang L. PRAS40 deregulates apoptosis in Ewing sarcoma family tumors by enhancing the insulin receptor/Akt and mTOR signaling pathways. Am J Cancer Res. 2016;6:486–97. 27186418 PMC4859675

[44] Qi Z, Zhang T, Song L, Fu H, Luo H, Wu J, et al. PRAS40 hyperexpression promotes hepatocarcinogenesis. EBioMedicine. 2020;51:102604. 31901857 10.1016/j.ebiom.2019.102604 PMC6950779

[45] Madhunapantula SV, Sharma A, Robertson GP. PRAS40 deregulates apoptosis in malignant melanoma. Can Res. 2007;67:3626–36. 10.1158/0008-5472.CAN-06-4234 17440074

[46] Godfrey JD, Morton JP, Wilczynska A, Sansom OJ, Bushell MD. MiR-142-3p is downregulated in aggressive p53 mutant mouse models of pancreatic ductal adenocarcinoma by hypermethylation of its locus. Cell Death Dis. 2018;9:644. 29844410 10.1038/s41419-018-0628-4 PMC5973943

[47] Abdul Razak SR, Baba Y, Nakauchi H, Otsu M, Watanabe S. DNA methylation is involved in the expression of miR-142-3p in fibroblasts and induced pluripotent stem cells. Stem Cells Int. 2014;2014:101349. 25544846 10.1155/2014/101349 PMC4269320

[48] Skarn M, Baroy T, Stratford EW, Myklebost O. Epigenetic regulation and functional characterization of microRNA-142 in mesenchymal cells. PLoS ONE. 2013;8:e79231. 24236112 10.1371/journal.pone.0079231 PMC3827369

[49] Lam HC, Siroky BJ, Henske EP. Renal disease in tuberous sclerosis complex: pathogenesis and therapy. Nat Rev Nephrol. 2018;14:704–16. 30232410 10.1038/s41581-018-0059-6

[50] Henske EP, Jozwiak S, Kingswood JC, Sampson JR, Thiele EA. Tuberous sclerosis complex. Nat Rev Dis Primers. 2016;2:16035. 27226234 10.1038/nrdp.2016.35

[51] Xiong X, Xie R, Zhang H, Gu L, Xie W, Cheng M, et al. PRAS40 plays a pivotal role in protecting against stroke by linking the Akt and mTOR pathways. Neurobiol Dis. 2014;66:43–52. 24583056 10.1016/j.nbd.2014.02.006 PMC4448971

[52] Di Malta C, Zampelli A, Granieri L, Vilardo C, De Cegli R, Cinque L, et al. TFEB and TFE3 drive kidney cystogenesis and tumorigenesis. EMBO Mol Med. 2023;15:e16877. 36987696 10.15252/emmm.202216877 PMC10165358

[53] Alesi N, Khabibullin D, Rosenthal DM, Akl EW, Cory PM, Alchoueiry M, et al. TFEB drives mTORC1 hyperactivation and kidney disease in tuberous sclerosis complex. Nat Commun. 2024;15:406. 38195686 10.1038/s41467-023-44229-4 PMC10776564

