# Supplementary material for: mTOR/miR-142-3p/PRAS40 signaling cascade is critical for tuberous sclerosis complex-associated renal cystogenesis
Source: Cell Mol Biol Lett. 2024 Sep 27;29:125. doi: 10.1186/s11658-024-00638-x (PMC11429883; doi:10.1186/s11658-024-00638-x)
Supplement: Supplementary file 1 — Additional file 1. [file 11658_2024_638_MOESM1_ESM.docx]

**mTOR/miR-142-3p/PRAS40 signaling cascade is critical for tuberous sclerosis-associated renal cystogenesis**

Shuyun Zhao^1^*, Shuai Hao^1^*, Jiasheng Zhou^1^*, Tianhua Zhang^1, 2^, Zhaolai Qi^1^, Ting Zhang^1, 2^, Sajid Jalal^1^, Xinran Chen^1^, Chuanxin Zhai^1^, Lu Yin^1^, Yufei Bo^1^, Hongming Teng^1, 2^, Hongbing Zhang^3^, Lin Huang^1, 2^

**Supplemental materials**


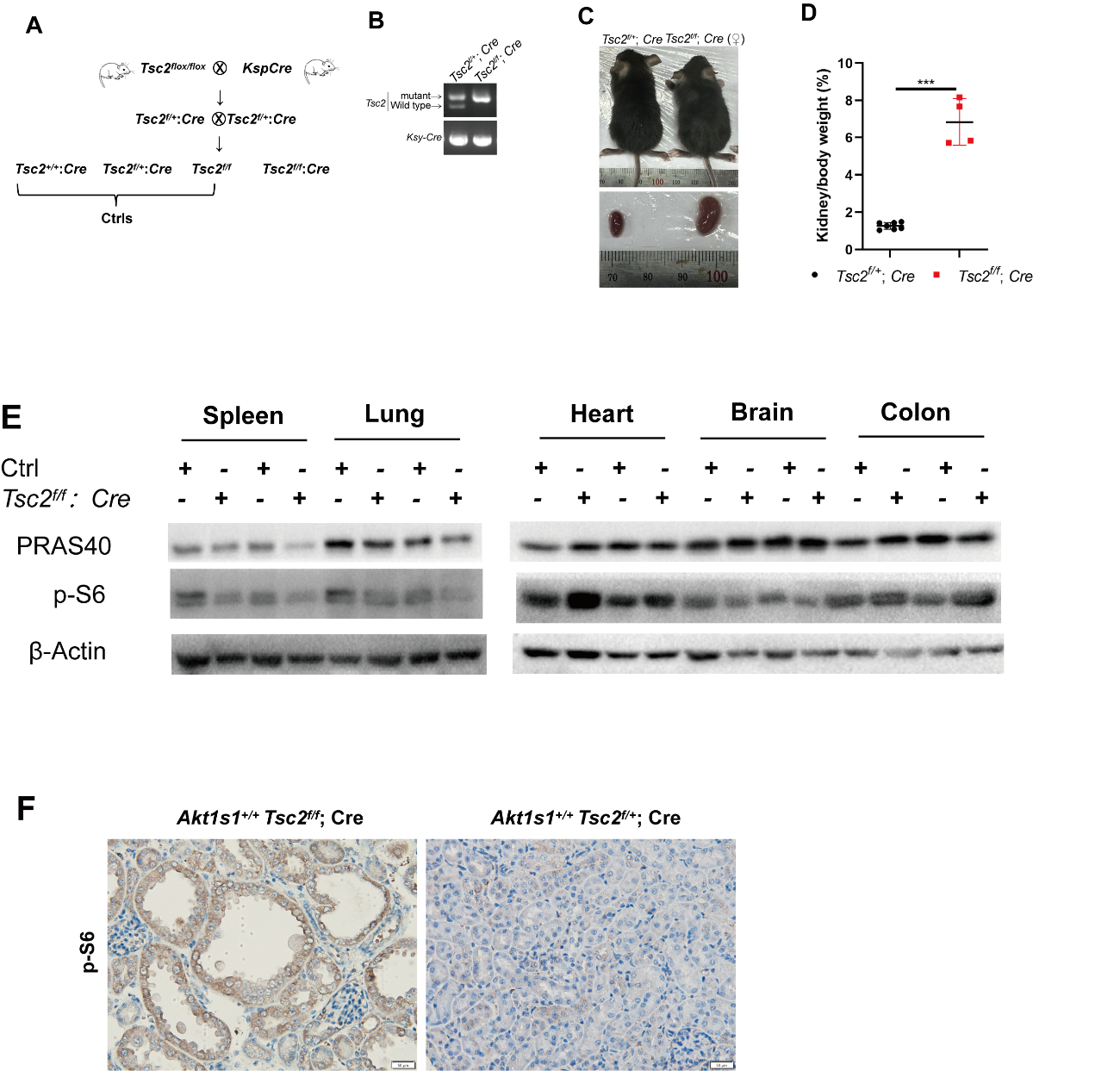


Figure S1. The alteration of *Tsc2*^f/f^*:* *ksp cre* mice.

A. Crossing strategy of the mice. B. Genotyping results. C. The images of the 20-day-old mice and the kidneys. D. The ratios of kidney weight to body weight (n=7 for *Tsc2*^f/+^; *Cre* and n=4 for *Tsc2*^f/f^; *Cre* mice). E. Western blotting. F. IHC staining. Bars, SD. ***, *P*<0.001 from Student’s t-test.


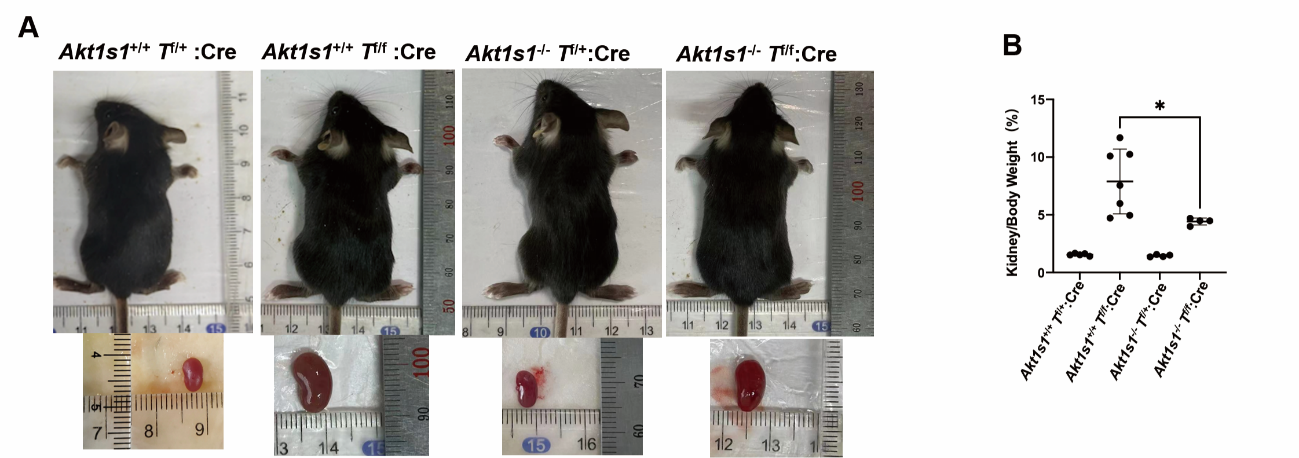


Figure S2. The difference between the kidneys of *Akt1s1^-/-^Tsc2*^f/f^; *Cre* and *Akt1s1^+/+^Tsc2*^f/f^; *Cre* female mice.

A. The images of the 20-day-old female mice and the kidneys. C. The body weights of the mice. D. The percentages of kidney weight to body weight. n=5 for *Akt1s1^+/+^Tsc2*^f/+^; *Cre* mice; n=7 for *Akt1s1^+/+^Tsc2*^f/f^; *Cre* mice; n=4 for *Akt1s1^-/-^Tsc2*^f/+^; *Cre* and *Akt1s1^-/-^Tsc2*^f/f^; *Cre* mice. Bars, SD. *, *P*<0.05 from one-way ANOVA.
